# The Role of Exosomes in Medical Dermatology: Literature Review and Update

**DOI:** 10.1111/jocd.16761

**Published:** 2025-01-10

**Authors:** Victoria Dukharan, Milaan Shah, Luke Broughton, Carol Stegura, Luna Samman, Nina Schur, Jacqueline M. Luna, Daniela Gutierrez Mendoza, José Ramón García Lira, Adriana María Valencia‐Herrera, Jona Louise D. Macaraeg‐Jimenez, Victoria G. Belo, Regina Gabrielle Gatan, Suparuj Lueangarun, Ana Cecilia Amador Ruiz, Karolina Ziemlewski, Zuramis Estrada Blanco, Todd Schlesinger

**Affiliations:** ^1^ Department of Dermatology Kansas City University – GME Consortium/Advanced Dermatology and Cosmetic Surgery Orlando Florida USA; ^2^ Department of Dermatology Medical University of South Carolina Charleston South Carolina USA; ^3^ School of Medicine Medical University of South Carolina Charleston South Carolina USA; ^4^ Lake Erie College of Osteopathic Medicine Bradenton Florida USA; ^5^ Department of Dermatology Garnet Health Medical Center Middletown New York USA; ^6^ Belo Medical Group Philippines; ^7^ ARŌAH Center Tijuana Mexico; ^8^ Department of Dermatology National Institute of Health, Hospital Infantil de México Federico Gómez Mexico City Mexico; ^9^ Department of Aesthetic Medicine, College of Integrative Medicine Dhurakij Pundit University Bangkok Thailand; ^10^ Division of Dermatology DeMed Clinic Center Bangkok Thailand; ^11^ Mexican Social Security Institute, Family Medicine Unit 9 Mexico City Mexico; ^12^ Kliniki Ziemlewski Wroclaw Poland; ^13^ CIMEG Madrid Medical Center Madrid Spain; ^14^ Clinical Research Center of the Carolinas Charleston South Carolina USA

**Keywords:** acne, atopic dermatitis, exosomes, lichen simplex chronicus, psoriasis, radiation dermatitis, stem cells, systemic lupus erythematosus, vitiligo, vulvar sclerosis, wound healing

## Abstract

**Background:**

Exosomes are extracellular vesicles, composed of a phospholipid bilayer, that are primarily derived from stem cells. The contents of exosomes can be incorporated into the tissue in which they are introduced, which presents a unique therapeutic option.

**Aims:**

Exosomes have been investigated as a treatment for a number of medical ailments, but the literature supporting these indications is inconclusive. In addition, much of the study on exosomes and their uses has been recently completed. Thus, this review summarizes the efficacy and implications of exosomes in the treatment of different dermatologic conditions.

**Methods:**

A literature review surrounding the use of exosomes for multiple medical dermatological conditions was conducted. Additionally, we present numerous practical cases in which patients had been treated with exosomes.

**Results:**

Overall, the success of exosomes in treating medical dermatologic conditions demonstrated varying efficacy in the literature, but the preliminary evidence is generally positive. The patient cases also showed satisfactory clinical outcomes but further studies and cases will be necessary to fully characterize the efficacy of exosomes and the ideal modalities for their application, including formulation, mode of distribution, and frequency of treatment.

**Conclusions:**

Exosomes may serve as an effective treatment option for wound healing, reconstruction of skin flaps, radiation dermatitis, acne vulgaris, psoriasis, atopic dermatitis, allergic contact dermatitis, lichen simplex chronicus, vulvar lichen sclerosis, systemic sclerosis, systemic lupus erythematosus, and vitiligo although additional studies are needed to confirm their efficacy and safety.

## 1 Introduction

Exosomes are nano‐sized extracellular vesicles that are transported between numerous cell types to facilitate intercellular communication [1, 2, 3, 4]. These novel cell‐free therapeutics are composed of a plasma membrane‐derived phospholipid bilayer, which serves to protect their contents from enzymatic degradation. Exosomes are naturally secreted by various cell types, including mesenchymal stem cells (MSCs), epithelial cells, dendritic cells, and platelets, and can also be derived from cell cultures under controlled laboratory conditions. Their cargo includes a diverse array of bioactive molecules, such as proteins, lipids, mRNA, and microRNA, that can modulate the physiological and pathological states of recipient cells [5, 6].

The production of exosomes involves isolating the vesicles from biological fluids (e.g., blood, urine, or saliva) or conditioned media from cultured cells. The process typically begins with cell culture, where donor cells are cultivated under optimized conditions to enhance exosome secretion. The conditioned media containing exosomes are collected and purified by either differential ultracentrifugation, size‐exclusion chromatography, ultrafiltration, or polymer‐based precipitation. Advanced methods, such as tangential flow filtration and immunoaffinity capture, may also be used to ensure high purity and yield. Characterization of exosomes involves particle sizing, surface marker analysis, and cargo profiling, employing techniques such as nanoparticle tracking analysis (NTA), transmission electron microscopy (TEM), and flow cytometry [1, 3].

The application of exosomes, particularly those derived from MSCs, in various disease processes has attracted attention from both researchers and clinicians [7]. Their anti‐inflammatory and immunomodulatory properties make them appealing therapeutic candidates across diverse fields, including cardiovascular injury, cancer, kidney injury, aging, hypoxia‐induced myocyte injury, inflammatory bowel disease, and traumatic brain injury. Furthermore, exosomes are increasingly studied for their utility in treating skin conditions [8, 9, 10, 11, 12, 13, 14, 15, 16]. Over the last decade, there has been a growing body of research investigating various dermatological applications of exosomes, suggesting their potential in skin regeneration, wound healing, and immune modulation [7, 10, 17].

Given the rapid advancements and expanding interest in exosome‐based therapeutics, we aim to evaluate and describe the current literature and relevant clinical cases regarding their medical indications, with a particular focus on dermatology (Table 1). All clinical case photos presented utilize Exosome Smart Cleaning and Refining Technology (ExoSCRTTM ) a patented technology that enables the large‐scale production and purification of exosomes with high reproducibility and purity developed with ExoCoBioTM using adipose tissue and Damask rose stem cells. [Correction added on July 16, 2025, after first online publication: Additional details regarding the production of the exosomes used in the cases were added.] Additionally, written informed consent was collected from all patients in the included cases. The authors confirm that the ethical policies of the journal, as noted on the journal's author guidelines page, have been adhered to. No ethical approval was required as this is a review article with no original research data.

**TABLE 1:** Summary of exosome applications and outcomes in dermatology.

Condition	Exosome source	Application method	Outcome	Key study/Case description
Wound healing	ADSCs, iPSC‐derived	Topical	Accelerates keratinocyte/endothelial migration, reduces inflammation, and enhances re‐epithelialization	**Key Study:** Complete re‐epithelialization in animal models [18, 19, 20, 21, 22]
	Plant‐derived	Topical	Complete closure of chronic nonhealing axillary wound in 7 weeks	**Clinical Case:** 24‐year‐old female with hidradenitis suppurativa
	Plant‐derived	Topical	90% closure of a third‐degree ulcer with exposed muscle/bone in 26 days	**Clinical Case:** 79‐year‐old female with traumatic scalp injury
	Plant‐derived + Hyaluronic Acid Gel	Topical	Significant oral re‐epithelialization in 24 h, enabling oral feeding; lip healing by day 6	**Clinical Case:** 16‐year‐old female with Stevens–Johnson syndrome
Flap Reconstruction	ADSCs	Intradermal Injection	Improves skin flap survival rate, reduces scarring, promotes angiogenesis, and stimulates collagen and elastin production	**Key Study:** Animal studies demonstrate efficacy [23, 24, 25, 26]
Radiation Dermatitis	ESC‐derived, MSC‐derived, plasma‐derived	Topical, Intravenous	Exosomal miRNAs (e.g., miR‐126, miR‐135a, miR‐146) promote epithelial repair, protect against oxidative stress, and regulate immune responses	**Key Study:** Studies highlight the roles of miRNAs in epithelial migration, inflammation reduction, and antioxidant defense [27]
	Plant‐derived	Topical	Lightening of radiation‐induced pigmentation in 3 weeks; additional lightening of surgical scars	**Clinical Case:** 48‐year‐old female postradiation therapy for breast cancer
Acne Vulgaris	ADSCs	Intralesional	Inhibits inflammation (via reduced IL‐1β and NLRP3 inflammasome) and NETs, reducing acne severity	**Key Study:** Reduced inflammation and redness in mouse models [29, 30]
	Plant‐derived	Intralesional with air dissector, RF microneedling, and a combined 589 nm and 1319 nm laser	Significant lesion improvement after two sessions over 2 months. Therapy was administered for four sessions every 2 weeks	**Clinical Case:** 43‐year‐old male with severe acne vulgaris
	Plant‐derived	Topical with picosecond laser	Accelerated postlaser scar healing; reduced redness and swelling within a week compared with laser treatment without exosomes	**Clinical Case:** 25‐year‐old female with acne scars
	Plant‐derived	Topical with picosecond laser	Enhanced healing of acne scars 3 months after a single session of picosecond laser compared to ceramide moisturizer	**Clinical Case:** Comparison in a single patient postlaser treatment
Psoriasis	MSC‐derived, siRNA‐loaded	Topical	Reduces IL‐17/IL‐23 levels, suppresses immune dysregulation, and decreases scaling and erythema	**Key Study:** Reduced lesions and scaling in murine models [31, 32]
	Plant‐derived	Topical	Reduced scaling by 35 days and resolved erythema by 65 days	**Clinical Case:** 30‐year‐old female with erythematous plaques
Atopic Dermatitis (AD)	ADSCs	Topical, SubQ, IV	Reduces inflammatory cytokines, eosinophils, IgE levels; improves skin hydration and barrier function	**Key Study:** Reduced inflammation and improved hydration in mouse models [36, 37, 38]
	Plant‐derived	Topical	Complete clearance of refractory lesions in 21 days, with no recurrence after 2 months	**Clinical Case:** 6‐year‐old female with refractory AD
	Plant‐derived	Topical	Reduced facial redness caused by dupilumab therapy; increased patient satisfaction	**Key Study:** 12‐week prospective study on dupilumab‐associated facial redness [39]
Allergic Contact Dermatitis (ACD)	MSC‐derived	Topical	Modulates T‐cell responses, reduces proinflammatory cytokines, and increases Tregs and IL‐10	**Key Study:** Immune modulation demonstrated in mouse models of ACD [41]
	Plant‐derived	Topical	Resolution of pruritus, scaling, and redness in 14 days	**Clinical Case:** 65‐year‐old male with exfoliative dermatitis due to ACD
Lichen Simplex Chronicus (LSC)			Limited preclinical data on exosome therapy in LSC	
	Plant‐derived	Topical with microneedling	Reduced erythema, lichenification, and pruritus within 7 days; improved quality of life	**Clinical Case:** 39‐year‐old male with LSC
Vulvar Lichen Sclerosus (LS)			Limited preclinical data on exosome therapy in vulvar LS	
	Plant‐derived	Topical with RF Microneedling	Resumed sexual activity and reduced pruritus and atrophy after two sessions	**Clinical Case:** 60‐year‐old female with severe vulvar LS
Systemic Sclerosis (SSc)	MSC‐ derived, Umbilical cord‐derived	Intravenous	Reduces fibrosis, promotes immune balance, and improves macrophage polarization	**Key Study:** miRNA‐loaded exosomes reduce fibrosis via IL4Rα/mTOR pathway in murine studies [46, 47]
			No clinical cases reported yet	
Systemic Lupus Erythematosus (SLE)	Umbilical cord‐derived	Intravenous	Reduces inflammation (i.e., macrophage proliferation), expands regulatory T cells, and improves organ function	**Key Study:** Exosomes alleviate nephritis, liver damage, and cytokines in a murine study [51]
			No clinical cases reported yet	
Vitiligo	Umbilical cord‐derived, Keratinocyte‐derived	Intravenous, direct melanocyte transfection	Reduces CD8+ T cell infiltration and oxidative stress and promotes melanocyte survival and function	**Key Study:** Reduced depigmentation and enhanced melanocyte survival in mice [56, 63]

Abbreviations: ADSC, adipose‐derived stem cells; iPSC, induced pluripotent stem cells; MSC, mesenchymal stem cell; siRNA, small interfering RNA; SubQ, subcutaneous; IV, intravenous; RF, radiofrequency.

## 2 Exosomes in Wound Healing

There is significant evidence in the literature to support the role of exosomes in wound healing: studies by Bo et al. and Lee et al. using exosomes from human induced pluripotent stem cell–derived keratinocytes and adipose tissue–derived mesenchymal stem cells, respectively, have demonstrated that their topical administration accelerates wound healing through enhanced keratinocyte and endothelial cell migration [18, 19]. Similarly, exosomes play a role in modulating inflammation to promote wound healing, as demonstrated by complete re‐epithelialization of chronic wounds in rat and rabbit models [20, 21, 22].

**FIGURE 1:**
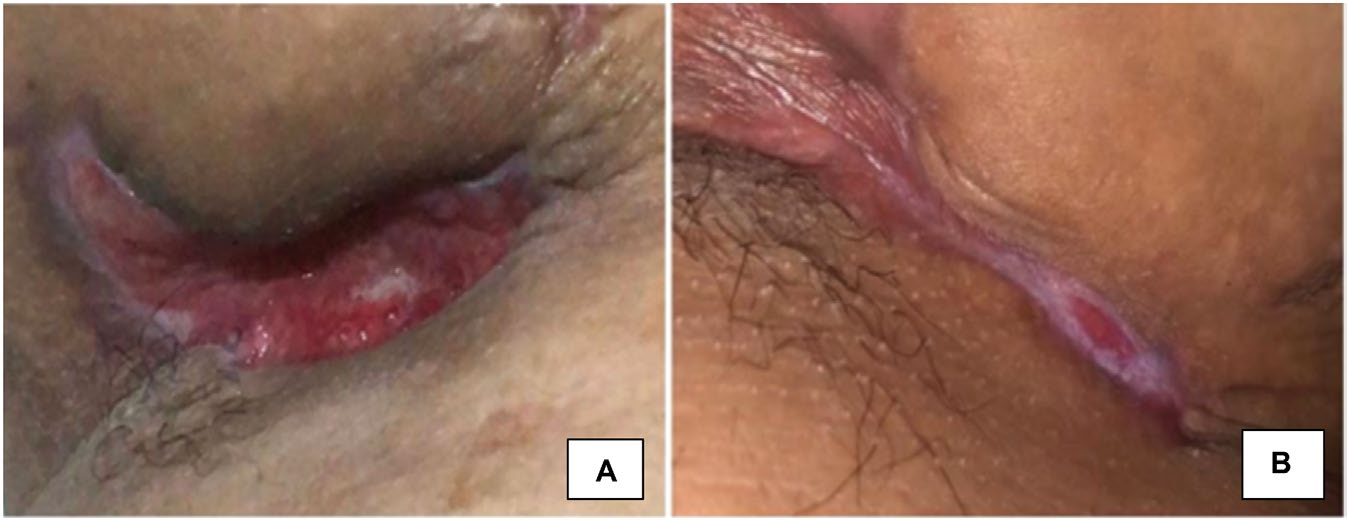
(A) Postsurgical wound of the patient in the right axilla which remained open for 8 months. (B) Complete closure of the wound after 7 weeks of topical exosome application. Images provided courtesy of Dr. Jacqueline Luna.

In the recent case of a 24‐year‐old female with hidradenitis suppurativa, exosomes proved to be pivotal in the resolution of nonhealing surgical wounds. In the right axilla, she had a wound which persisted for 8 months after surgical sinus tract removal which was left to heal by secondary intention (Figure 1A). During this time, the patient had used systemic antibiotics as well as daily topical antibiotics, but no healing was observed. Complete closure of this wound was seen after 7 weeks of twice daily topical application of a plant‐derived exosome solution developed by ExoCoBio (Figure 1B and Table 1).

**FIGURE 2:**
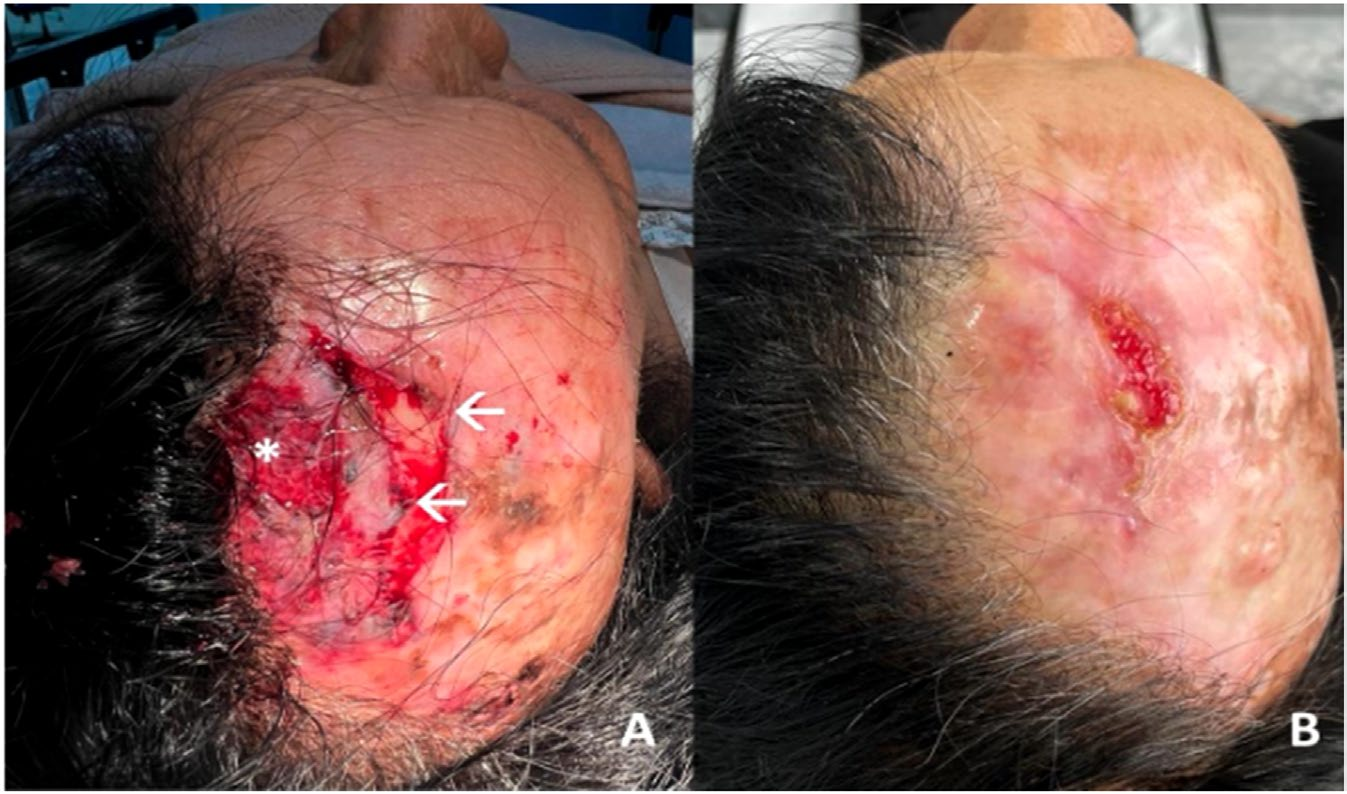
(A) Ulcer on day 0 with exposed muscle (*) and bone (→). (B) Day 26 after nine sessions of topical exosomes. Images provided courtesy of Dr. Daniela Gutiérrez Mendoza.

Exosomes also enhance wound therapy for patients who may require alternative treatments. A 79‐year‐old woman with a history of traumatic injury on the scalp was successfully treated with a full‐thickness graft. Forty years after her initial injury, she presented with a third‐degree traumatic ulcer due to reinjury at the graft site (Figure 2A). On initial evaluation, muscle and bone were exposed. Given the patient's poor skin quality and comorbidities, including arterial hypertension, diabetes, and history of prior graft at the site, surgical therapy such as repeat skin graft had a poor prognosis for recovery. Alternatively, topical exosome therapy was pursued. Prior to the initiation of exosomes, the patient completed a 5‐day course of IM ceftriaxone. The patient experienced 90% wound closure after nine applications of topical exosomes in 26 days, at intervals of 2–4 days (Figure 2B). The exosome solution was applied in combination with a hydrocolloid or alginate patch, depending on the level of wound exudate. This case demonstrates the efficacy of exosomes as an alternative to surgery for patients who are poor surgical candidates and for whom direct or graft closure is not possible.

**FIGURE 3:**
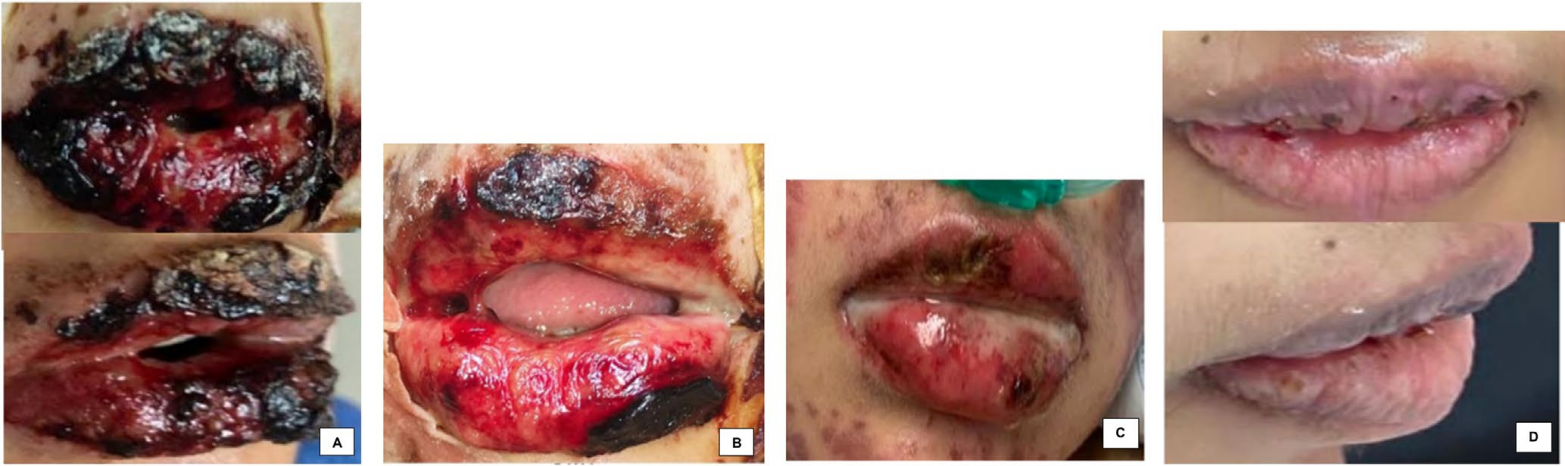
(A) Day 0 (baseline). (B) Day 2 (After the fourth session). (C) Day 4 (After the eighth session). (D) Day 6. Images provided courtesy of Dr. Ramón García.

Another recent case involves a 16‐year‐old female with Stevens–Johnson syndrome who presented with severe oral mucositis which prohibited oral feeding. She received comprehensive treatment with immunoglobulin, steroids and analgesia. However, due to persistent mucosal ulceration and hemorrhagic lip crusting, an adjuvant topical therapy was initiated. [Correction added on July 16, 2025, after first online publication: Additional details regarding the course of the clinical case were added.] An exosome solution (1 mL) plus 2% hyaluronic acid gel (4 mL) was applied to the oral cavity mucosa and lips every 12 h for 4 days. Despite her initial disease severity, just 24 h after the first application, she experienced considerable oral re‐epithelialization, which allowed feeding by mouth. Lip re‐epithelialization was adequate by the fourth day, and satisfactory results were seen on the sixth day (Figure 3). For patients with Stevens–Johnson syndrome, a therapy that accelerates the repair process can contribute to reduced morbidity and allow for earlier resumption of oral intake. Decreasing morbidity may also lead to less days of analgesics, reduced sepsis risk, and shorter hospital stay. Further investigation is necessary to determine if exosomes may be able to provide this benefit.

## 3 Exosomes in the Reconstruction of Skin Flaps

The use of exosomes in skin flap reconstruction has demonstrated promising results in animal studies conducted on rats [23]. Additionally, injection of adipose mesenchymal stem cell–derived exosomes into a skin flap has been found to significantly improve flap survival rate and reduce scarring [24, 25]. Injection of exosomes into skin promotes angiogenesis and stimulates the production of collagen and elastin, all of which are essential components in skin regeneration [26]. Current research on the impact of exosomes used in skin flap reconstruction is lacking, and further research conducted in human models is needed.

## 4 Exosomes in Radiation Dermatitis

Radiation‐induced skin injuries, including acute effects like erythema and desquamation and chronic effects like fibrosis and malignancy, result from the skin's high cell turnover and sensitivity to radiation. Exosomes play a crucial role in mitigating these injuries by promoting epithelial regeneration, reducing inflammation via miRNAs (e.g., miR‐126 and miR‐146), protecting against oxidative stress, and modulating immune responses, highlighting their potential in accelerating wound healing and delaying skin aging [27].

**FIGURE 4:**
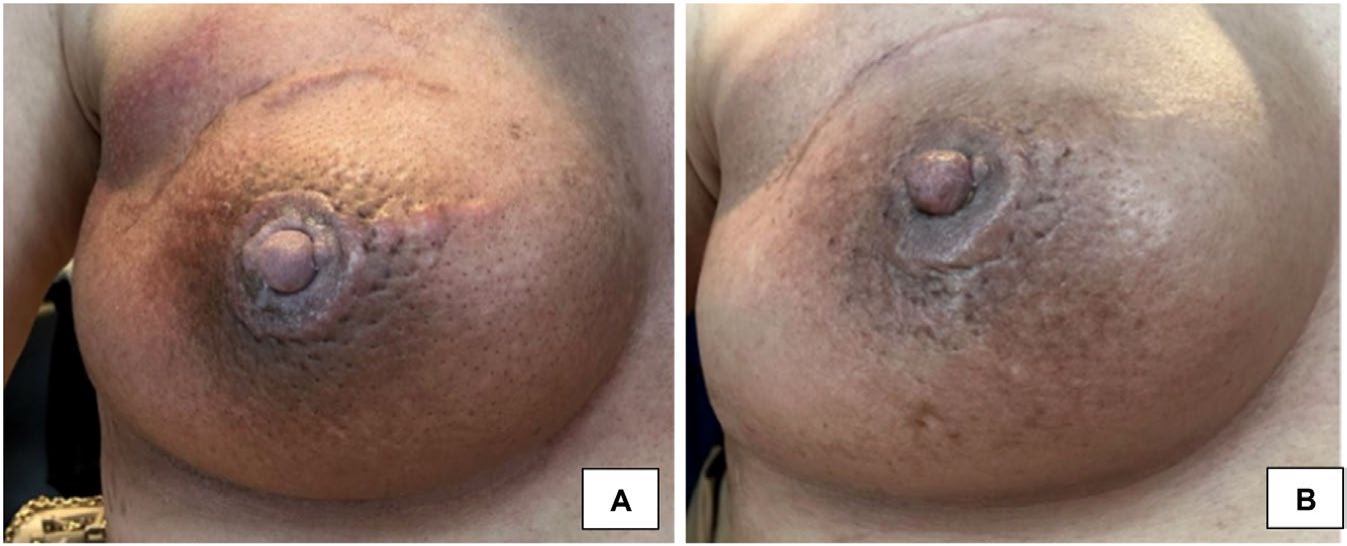
(A) Baseline image of the right breast with periareolar erythematous to brown patches. (B) After 3 weeks of twice daily applied exosomes, lightening of previously affected skin is observed. Images provided courtesy of Dr. Jona Louise Macaraeg‐Jimenez.

The immunomodulatory and healing effects of exosome therapy have been demonstrated in a case of postradiation dermatitis treatment. A 48‐year‐old female diagnosed with invasive carcinoma of the right breast underwent lumpectomy and radiation treatment for thirty consecutive days. She noted that radiation therapy caused dark brown pigmentation to develop around the nipple–areola complex of the affected breast. One day after her radiation therapy ended, topical plant‐derived exosome applications were started. The formula was applied twice daily for 3 weeks. Improvement in lightening was observed throughout the previously affected periareolar area (Figure 4). Incidentally, the patient also noted lightening of the surgical scar on the right upper breast. Eight years prior, the patient underwent the same treatment regimen with lumpectomy and radiation on the same breast. At that time, she had applied emollients to the hyperpigmented area with lightening of the skin seen after 2–3 months. In this case, the use of an exosome‐based topical therapy produced rapid results within only 3 weeks of treatment compared to several months previously. There were no significant adverse events. Further studies should be conducted to understand the efficacy of topical exosomes compared to other accepted hypopigmenting agents such as hydroquinone, retinoids, kojic acid, and other commonly used topicals.

## 5 Exosomes in Acne Vulgaris

Acne vulgaris is a very common skin disease with several available treatment options. For severe acne or acne resistant to topical treatments, oral isotretinoin may be prescribed. Given its high teratogenicity and mucocutaneous symptoms, isotretinoin is not a viable treatment for all patients [27]. Exosomes present an effective, alternative therapy.

Exosomes secreted by adipose‐derived stem cells (ADSCs) have primarily been studied in the literature. To examine the impacts of exosomes, Yu et al. [28] used a mouse model where the stem cells were incubated with anti‐CD29, ‐CD44, ‐CD90, and ‐CD105 as positive markers for ADSCs. Three separate groups of mice were used, one group was inoculated with *C. acnes*, the bacteria known to cause acne in humans, another group inoculated with *C. acnes* and ADSCs, and finally, a control group was inoculated with PBS. Upon examination of photographs between the groups, there was significantly less redness noted in the mice group that received ADSC treatment compared to the group that only received *C. acnes* inoculation. Furthermore, the thickness of the auricle and of the stratum corneum were decreased in the treatment group. Infiltration of neutrophils in the stromal layer of the skin was also decreased in the treatment group when compared to the group receiving only bacterial inoculation. The results of this study suggest that ADSCs not only reduce neutrophil extracellular trap (NET), but that they reduce hyperkeratosis, both of which are important pathways in the pathogenesis of acne vulgaris.

Another group examined the impact of ADSCs on the nod‐like receptor family pyrin domain‐containing 3 (NLRP3) inflammasome using a similar mouse model with four groups, one given only PBS, one inoculated with *C. acnes*, another with *C. acnes* and PBS, and a final group inoculated with *C. acnes* and ADSCs [29]. This group also found that ADSCs were effective in reducing inflammation, particularly by inhibiting NLRP3 activation by suppressing caspase‐1 and therefore IL‐1β expression. As a result, mice in the treatment group receiving ADSCs had much lower levels of inflammation when compared to mice only inoculated with *C. acnes*.

**FIGURE 5:**
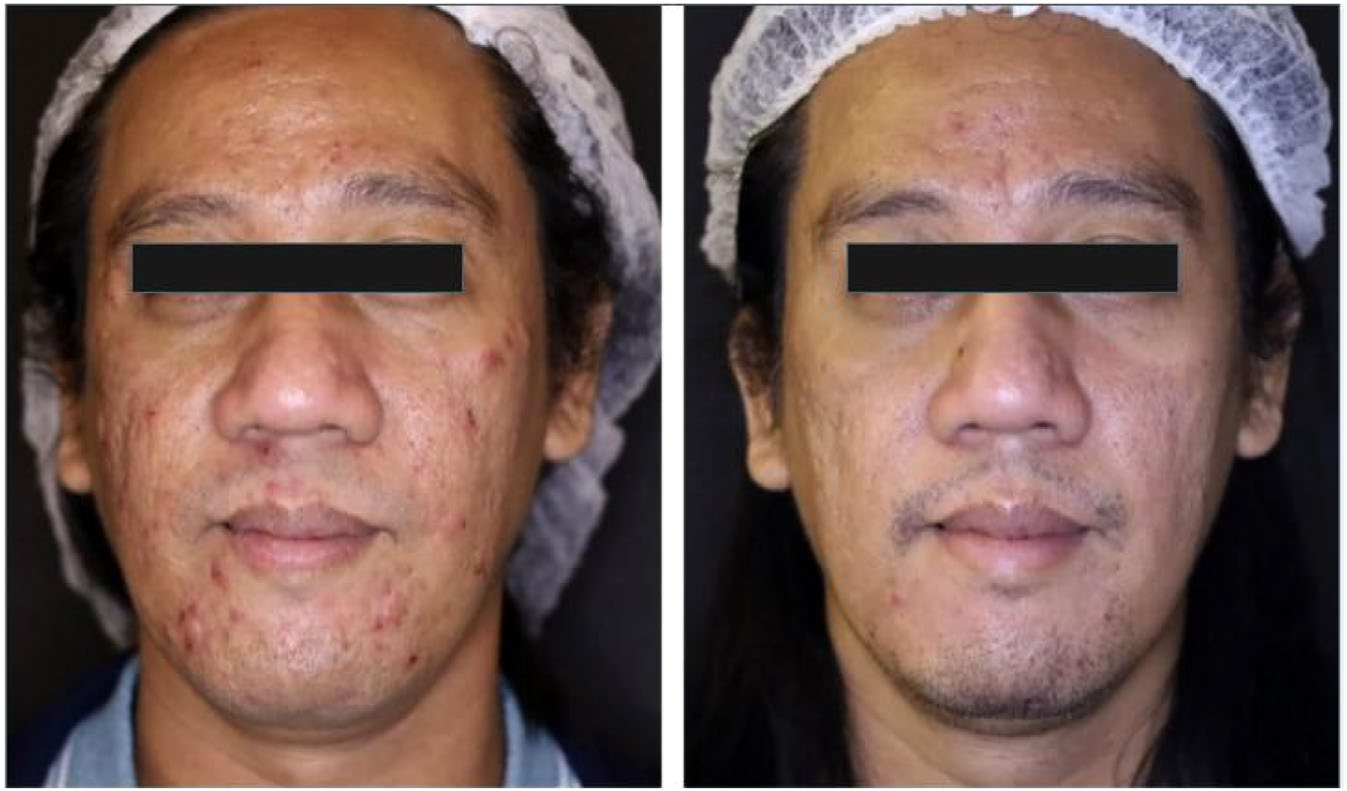
Before exosome treatment (left) and after exosome treatment (right). Images provided courtesy of Dr. Regina Gabrielle Gatan.

**FIGURE 6:**
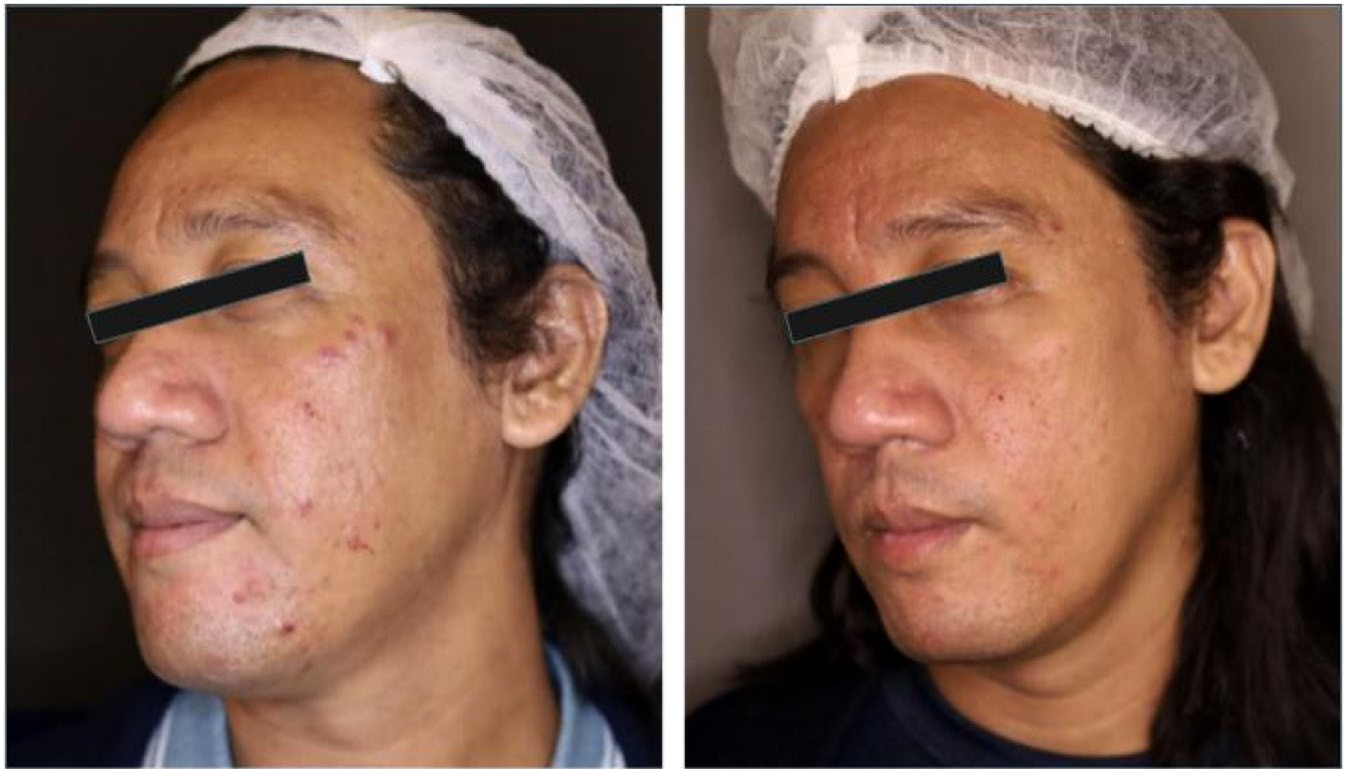
Before exosome treatment (left) and after exosome treatment (right). Images provided courtesy of Dr. Regina Gabrielle Gatan.

**FIGURE 7:**
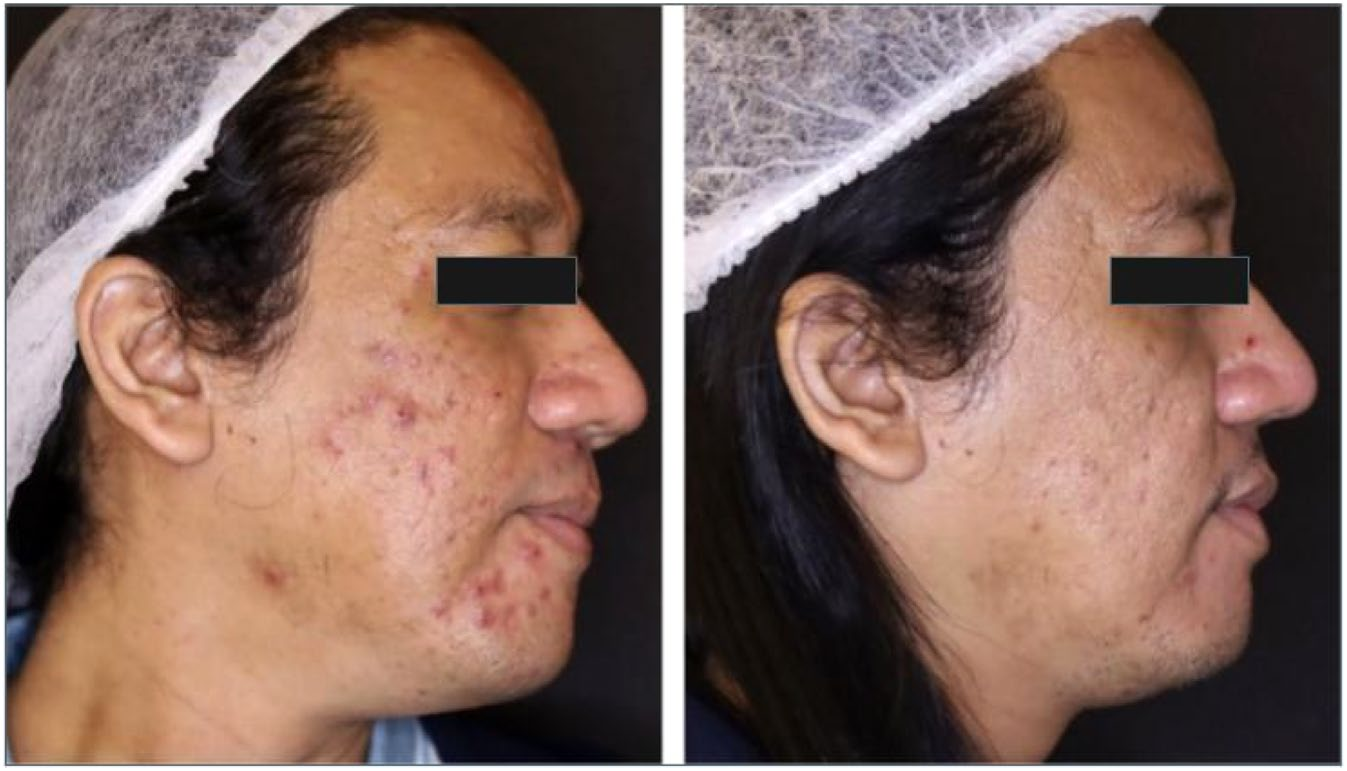
Before exosome treatment (left) and after exosome treatment (right). Images provided courtesy of Dr. Regina Gabrielle Gatan.

In a recent case for the treatment of acne vulgaris, plant‐derived exosomes were administered by intralesional injection. The 43‐year‐old male patient in the study received his treatment once a month for 2 months. Treatment involved an air dissector, RF microneedling, intralesional exosomes, and a combined 589 and 1319 nm laser for four sessions every 2 weeks. The before photos can be seen on the left and the after on the right for the series of figures. Photos were taken roughly 2 months apart. The first figure is a front‐facing view (Figure 5), with the left (Figure 6), and right (Figure 7) side profiles are shown as well. The figures demonstrate a significant improvement in the patient's acne in a 2‐month period and suggest some support for the use of this therapy in those suffering with acne vulgaris.

**FIGURE 8:**
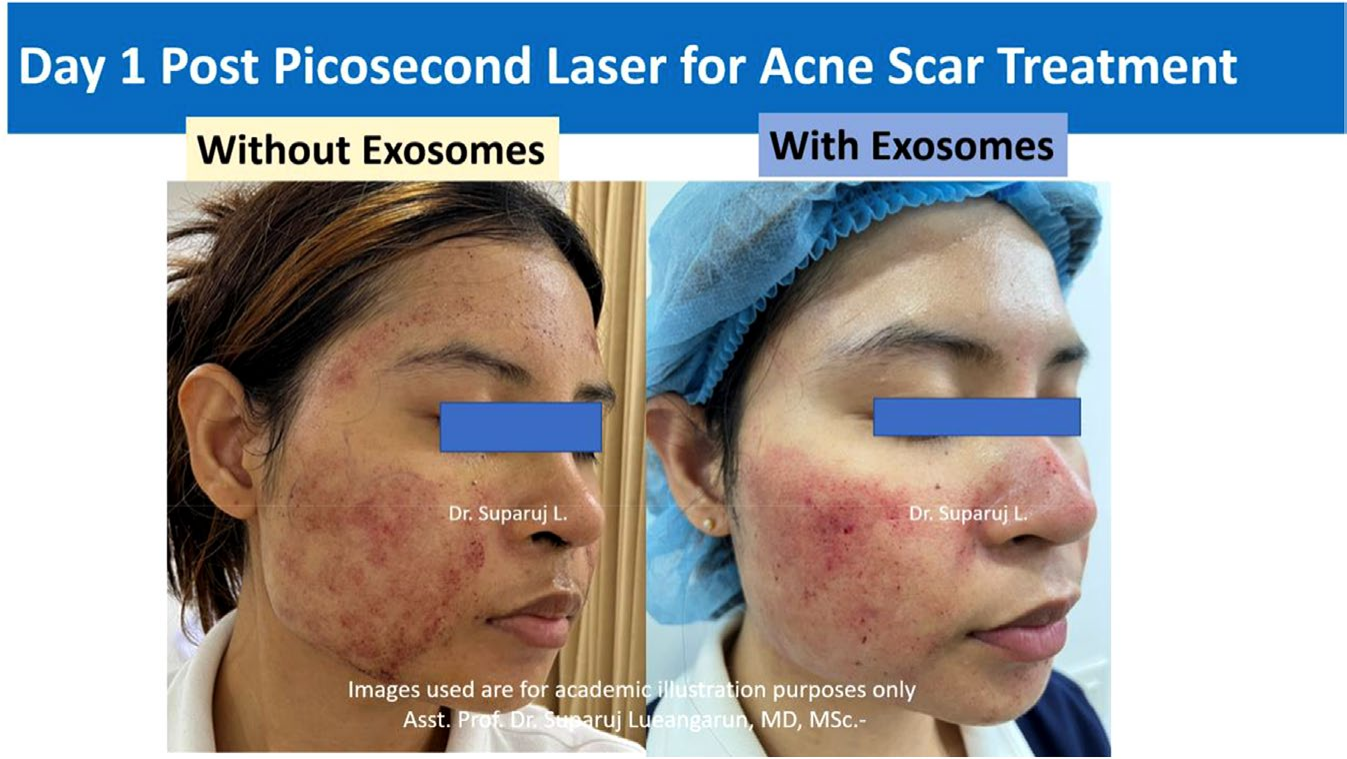
Side effects of laser treatment seen without (left) and with (right) exosome therapy on day 1 postlaser treatment. Images provided courtesy of Dr. Suparuj Lueangarun.

Another recent study found that the use of exosome therapy in conjunction with picosecond laser treatment significantly decreased the amount of time required for healing to occur in the treatment of acne scars. The patient in this study is a 25‐year‐old female with no prior treatment in the past 6 months. Exosome therapy on day 1 was found to be able to reduce the amount of redness, swelling, and level of inflammation from laser treatment when compared to laser treatment without exosomes (Figure 8).

**FIGURE 9:**
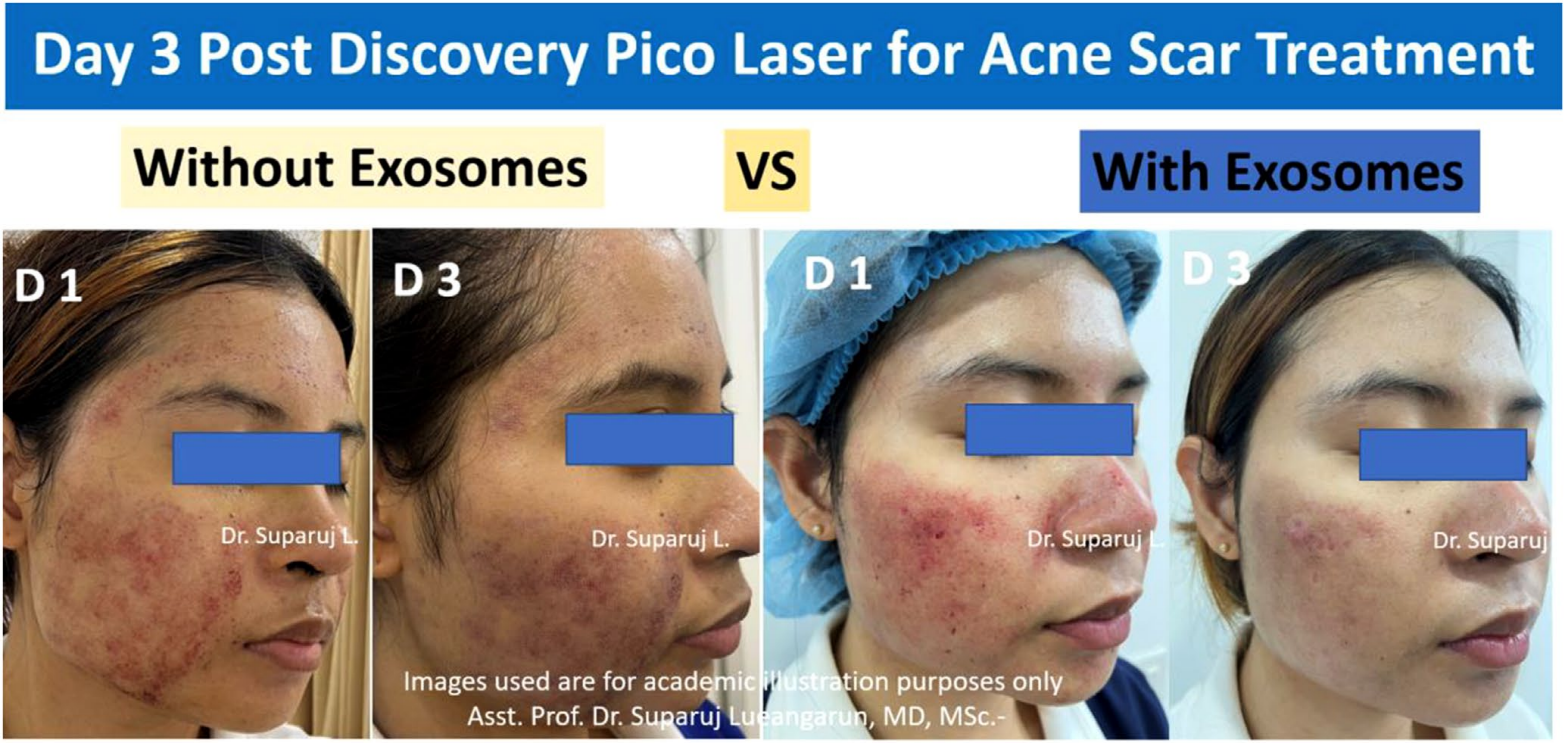
Comparing the side effects of laser treatment on days 1 and 3 posttreatment without (left) and with (right) additional exosome therapy. Images provided courtesy of Dr. Suparuj Lueangarun.

On day 3, exosome therapy continued to show significant benefit posttreatment (Figure 9).

**FIGURE 10:**
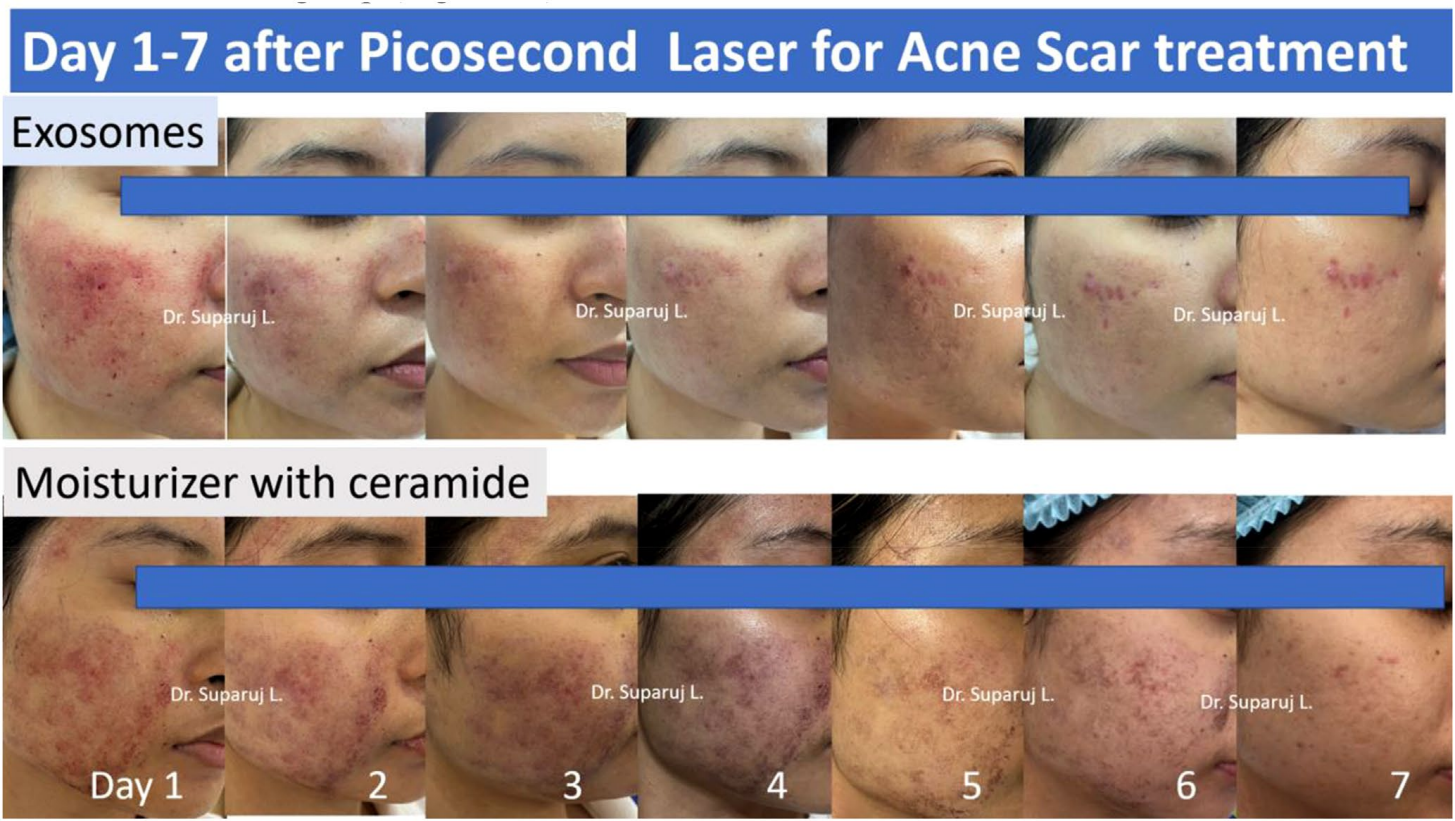
Comparing the side effects posttreatment of laser treatment on days 1 through 7 with (top) and without (bottom) additional treatment of exosomes. Images provided courtesy of Dr. Suparuj Lueangarun.

Benefits of exosome therapy continued to be seen through a week when compared to the nonexosome treatment group (Figure 10).

**FIGURE 11:**
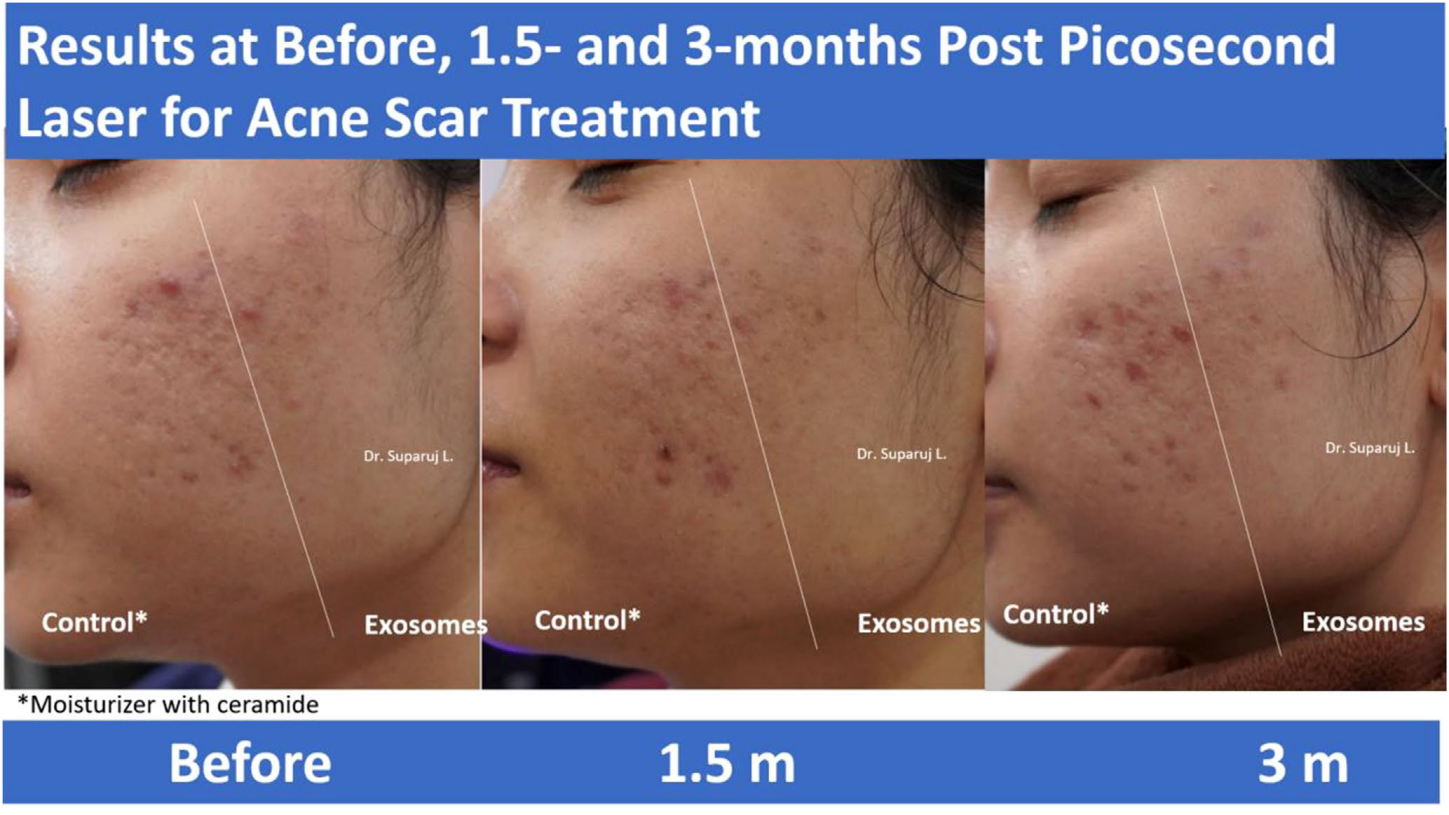
Comparing the efficacy of exosomes to a ceramide moisturizer for the capacity to accelerate scar healing in the setting of postpicosecond laser for acne scar treatment. Images provided courtesy of Dr. Suparuj Lueangarun.

The researchers also compared the impact of exosome therapy in scar treatment using only a single session. A ceramide moisturizer was used on the more medial aspect of the face while exosome therapy was used on the more lateral aspect (Figure 11). Pictures were taken for comparison before, at 1 month and a half, and at 3 months posttreatment.

The patient reported a satisfaction of 10 out of 10 with the use of exosomes in the acceleration of acne scar healing, supporting the use of this therapy for patients in the future with similar aesthetic complaints.

## 6 Exosomes in Psoriasis

Exosomes are believed to play a role in the overactive immune response which underlies psoriasis. Exosomes derived from regulatory T cells have been shown to reduce the production of IL‐17, IL‐23, and terminal complement complex (C5b‐9), in mice with a psoriasis‐like condition [30]. Similarly, exosomes loaded with small interfering RNAs (siRNAs) reduced skin lesions in a mouse model of psoriasis [31].

**FIGURE 12:**
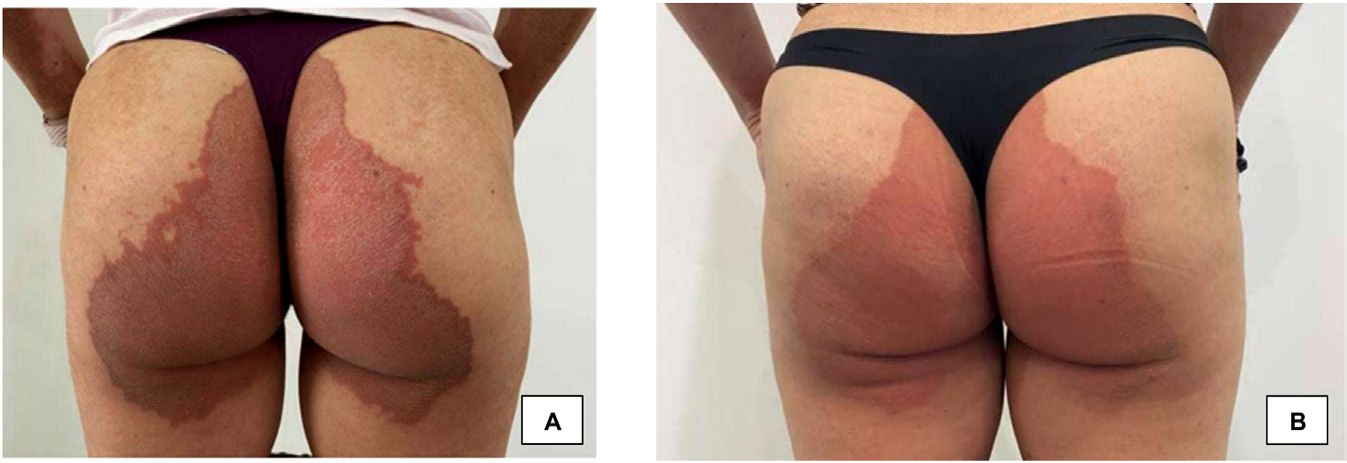
(A) Baseline image prior to therapy. (B) Thirty‐five days after first session. Images provided courtesy of Dra Ana Cecilia Amador Ruiz.

The utility of exosomes is demonstrated in a case of a 30‐old female who presented with erythematous, scaly plaques, disseminated to the arms, legs, thighs, and gluteus. She had a biopsy showing superficial perivascular dermatitis due to lymphocytes with hyperkeratosis, focal parakeratosis, hypergranulosis, and papillomatosis. She was treated with topical application of an exosome solution approximately once per month for a total of three sessions. Thirty‐five days after the first session, there was decreased scaling (Figure 12). Thirty days after the second session, the erythema resolved.

## 7 Exosomes in Atopic Dermatitis

Atopic dermatitis (AD) is a proinflammatory disease that can significantly affect patient quality of life due to intense pruritus [32]. Genetics and damage to the epidermal barrier are known to play a part in the pathophysiology of AD. Exosomes have been shown to decrease inflammation, promote angiogenesis, and stimulate fibroblasts [33, 34]. AD's pathophysiology is not completely understood, but the propensity of exosomes to decrease inflammation, a hallmark of AD, has been a notable indication for its use in treatment.

In a mouse model, exosomes derived from adipose stem cells were isolated and administered either intravenously (IV) or subcutaneously (SubQ) three times a week for 4 weeks. In both the IV and SubQ groups, exosomes caused a significant dose‐dependent decrease in AD symptoms. The number of CD86+ and CD206+ cells, which are uniquely expressed in AD lesions, were shown to decrease after administration of the exosomes. Serum IgE levels were similarly reduced after administration of exosomes and prednisolone. Exosomes were shown to significantly reduce the number of eosinophils and mRNA expression of inflammatory cytokines, including interleukin (IL)‐4, IL‐23, IL‐31, and tumor necrosis factor (TNF)‐α, in skin with lesions of AD in mice [35].

In another mouse model, extracellular vesicles isolated from adipose–derived stem cells from canines (cASC‐EVs) were applied to AD skin lesions created using Dermatophagoides Farinae ointment, and the clinical severity of AD was compared weekly. IgE was measured with an IgE ELISA kit. AD lesions were then removed from the backs of the mice, and mRNA levels of cytokines were measured with RT‐qPCR. The use of cASC‐EVs was found to decrease levels of serum IgE, inflammatory cytokines, and chemokines such as IL‐4 and interferon (IFN)‐γ [36].

Adipose tissue–derived exosomes have even been investigated for use in particulate matter‐induced inflammatory responses. Particulate matter has been studied and found to exacerbate AD. In a different study, ASC exosomes were examined for treatment of particulate matter–induced AD. Proinflammatory cytokines (IL‐6, IL‐1β, and IL‐1α) levels were explored using real‐time polymerase chain reaction, western blotting, and immunofluorescence. In the control group of mice with particulate matter AD, proinflammatory cytokines were increased, and anti‐inflammatory IL‐10 was decreased. Skin barrier proteins such as loricrin and filaggrin were also decreased. After application of ASC exosomes, these effects were reversed [37].

An additional study examining ASC exosomes and their effectiveness in spongiotic disorders had mice with oxazolone‐induced dermatitis receive subcutaneous injections of ASC exosomes. After receiving exosome treatment, the mice had reduced transepidermal water loss, increased stratum corneum hydration, and decreased levels of inflammatory cytokines. ASC exosomes also stimulated the creation of ceramides and dihydroceramides [32].

**FIGURE 13:**
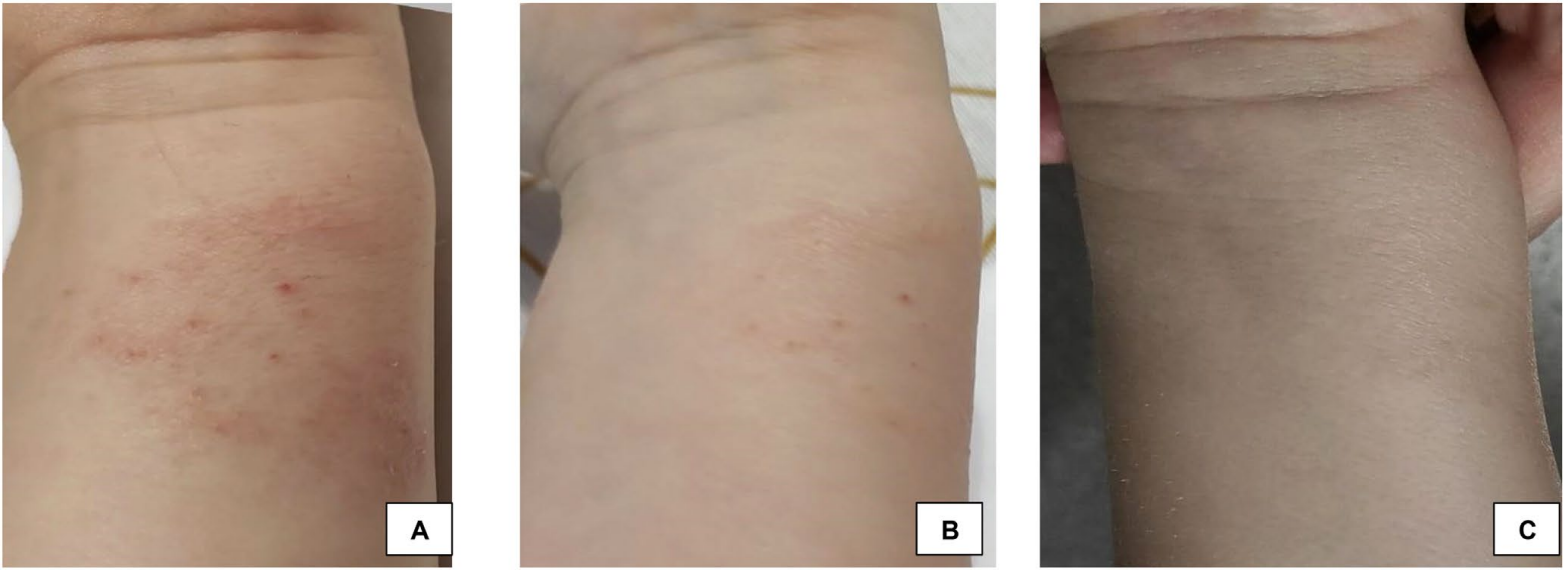
(A) Before exosome treatment. (B) Nine hours after the first session. (C) Twenty‐one days after the first session. Images provided courtesy of Dr. Karolina Pazera.

In a recent case, a 6‐year‐old female who experienced atopic lesions without improvement after many trials of different ointments received topical plant‐derived exosomes. After initial evaluation, the topical plant‐derived exosome solution was applied to her atopic lesions and examined after 15 h. The patient's AD began to clear 9 h after application, and complete clearance was seen after 21 days (Figure 13). After 2 months, the patient did not have a recurrence of atopy in any of the areas that were previously treated with topical exosomes.

Exosomes have also been used for the treatment of side effects of a dupilumab, a common and effective therapy for AD. A 12‐week prospective study examining if the use of topical exosomes could reduce facial redness after dupilumab use found that patient satisfaction was increased, and the erythema index decreased with use. Stratum corneum samples also showed that IL‐1α and human thymic stromal lymphopoietin were suppressed while filaggrin and vascular endothelial growth factor were stimulated [38].

## 8 Exosomes in Allergic Contact Dermatitis

Allergic contact dermatitis (ACD) is a skin condition which occurs as a result of an allergen‐specific T‐cell‐mediated inflammatory response [39]. Through immunoregulation, exosomes can affect the occurrence and development of ACD. As established in mouse models, exosomes can inhibit the development of Th1 and T cytotoxic type 1 (Tc1) cells and reduce the secretion levels of IL‐1β, TNF‐α, and IFN‐γ [40]. Exosomes also promote the expression of Treg cells and increase the levels of IL‐10, leading to enhanced regulation of the effector class of T cells and reduction in the autoimmunity response. Topical exosome therapy may provide a viable treatment option for ACD given its immunoregulatory properties.

**FIGURE 14:**
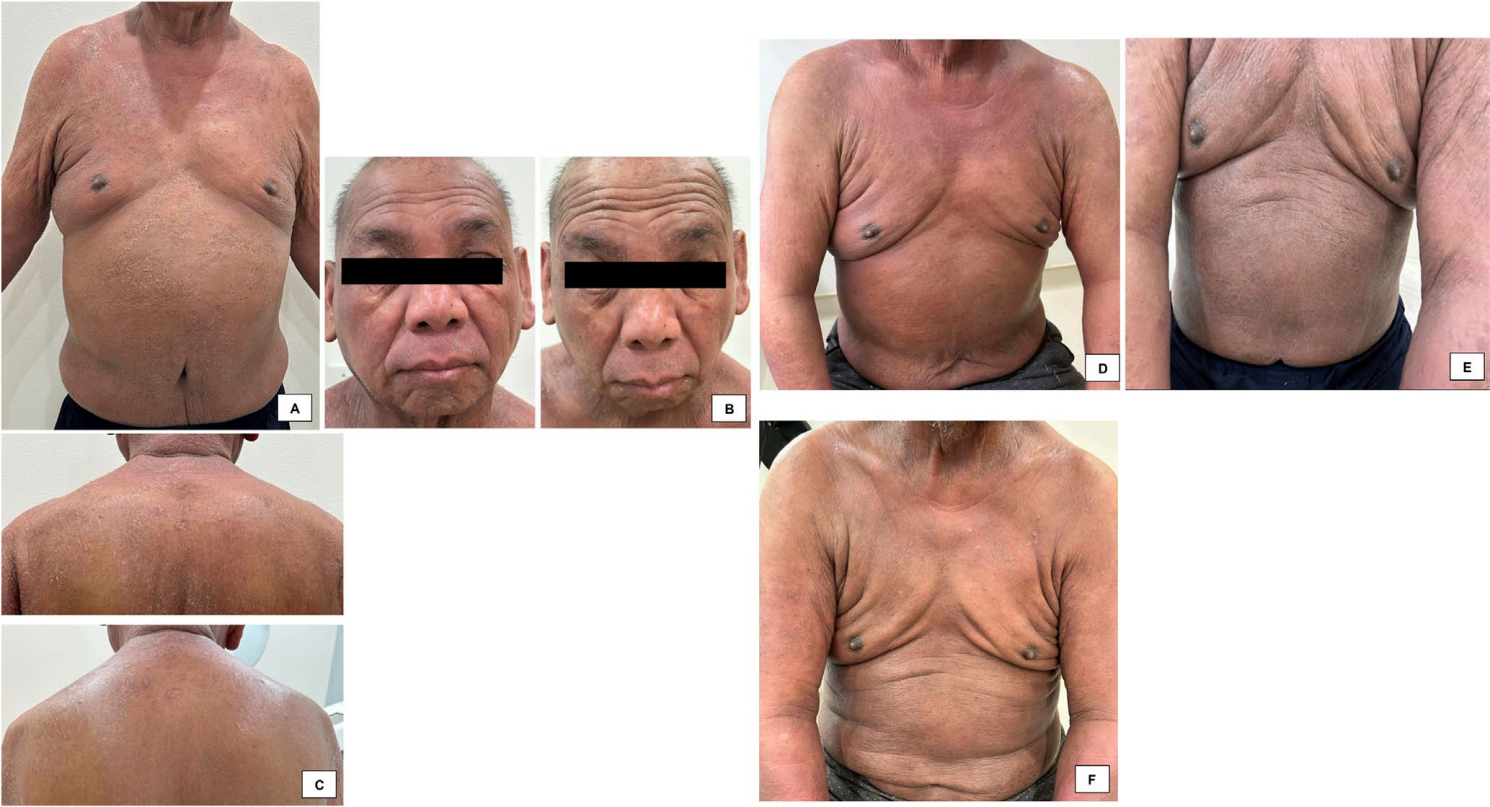
(A) Baseline image of exfoliative dermatitis prior to therapy. (B, C) Reduction in redness and scaling on the face and back 30 min after the first topical application of exosomes. (D) Three days after the first treatment session. (E) Three days after the second treatment session. (F) Fourteen days after the third treatment session. Images provided courtesy of Drs. Elaine Melody and Rosario Salud Abaya Blas.

The efficacy of exosomes was documented in a case of a 65‐year‐old male with exfoliative dermatitis due to ACD who experienced improvement in pruritus, scaling, and redness with treatment. The patient had used several topical agents including herbal creams and *aloe vera* gel prior to developing erythematous patches with subsequent exfoliation and intense itching. Initial treatments included oral antihistamines, topical steroids, and emollients. He also completed a course of oral clindamycin for cellulitis on the legs. Despite these treatments, the patient had minimal improvement after 2 weeks. A topical exosome solution was applied every 3 days for a total of three sessions. Simultaneously, the patient continued oral antihistamines and topical emollients. After three treatment sessions, the patient experienced symptomatic relief and resolution of lesions (Figure 14).

## 9 Exosomes in Lichen Simplex Chronicus

**FIGURE 15:**
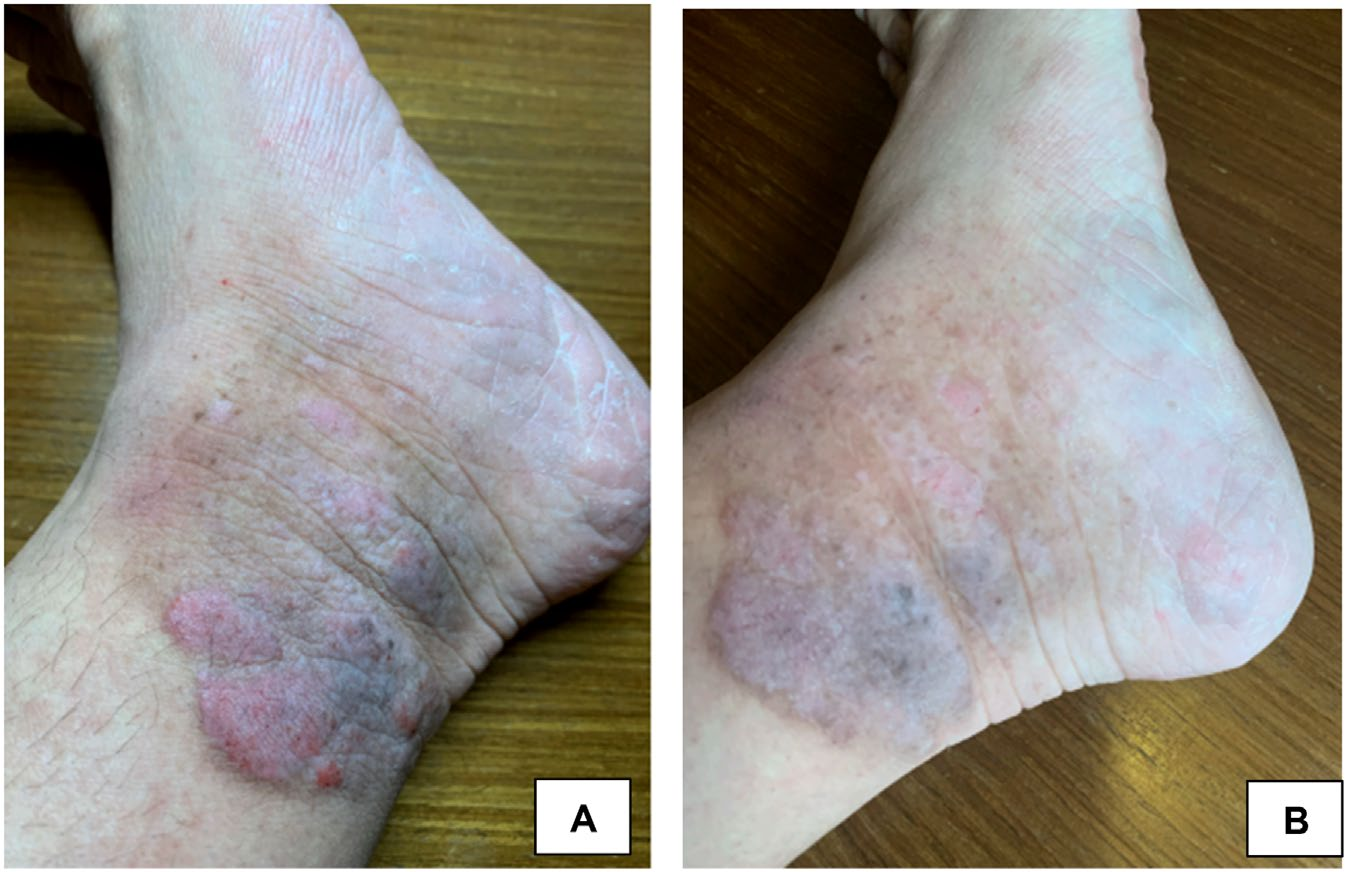
(A) Baseline image prior to treatment. (B) Seven days after one treatment session. Images provided courtesy of Dr. Rosario Salud Ramona A. Blas.

Lichen simplex chronicus (LSC) is regarded as a neurodermatitis which develops due to scratching. The condition may be primarily due to psychiatric comorbidities or secondary to other conditions such as eczema or psoriasis [41]. Over the years, our understanding of this condition involving localized pruritus as well as the itch–scratch cycle has expanded. Along with an improved understanding, several treatment options are being developed for this notoriously debilitating condition. One case which used topical exosome therapy on LSC demonstrated resolution of pruritus along with significant improvement in the appearance of the skin and the patient's quality of life. A 39‐year‐old male with LSC who had previously attempted oral antihistamines, topical steroids, topical urea 10%, topical salicylic acid, and pulse dye laser, and refused further intralesional steroid injections, was alternatively offered exosome therapy. One session was performed, including the application of one layer of topical plant‐derived exosome solution by ExoCoBio followed by an automated microneedling pen adjusted to a depth of 1.2–1.5 mm over the thickest areas. This cycle was repeated for a total of five times, utilizing a total of three‐fourths of the reconstituted vial. The remaining solution was sent home in an insulated bag with ice, and the patient was advised to continue application twice daily until the vial was consumed, within a maximum of 5–7 days while it is stored in the refrigerator. Seven days after treatment, the initial plaque appeared less erythematous with reduced hyperpigmentation and lichenification (Figure 15).

## 10 Exosomes in Vulvar Lichen Sclerosus

Lichen sclerosus (LS) is a chronic, inflammatory disorder, affecting genital and extragenital skin. Though the condition is seen in both men and women, it is more commonly observed in females [42]. Vulvar LS has an enormous impact on quality of life, causing itch, pain, dysuria, restriction of micturition, dyspareunia, and significant sexual dysfunction. Longstanding uncontrolled disease is associated with an increased risk for squamous cell carcinoma of the vulva. Given the significant impact of uncontrolled vulvar LS on quality of life as well as the risk for malignancy, effective treatment is necessary. One valuable addition to our treatment armamentarium may be the use of exosomes, as exhibited by the following case.

**FIGURE 16:**
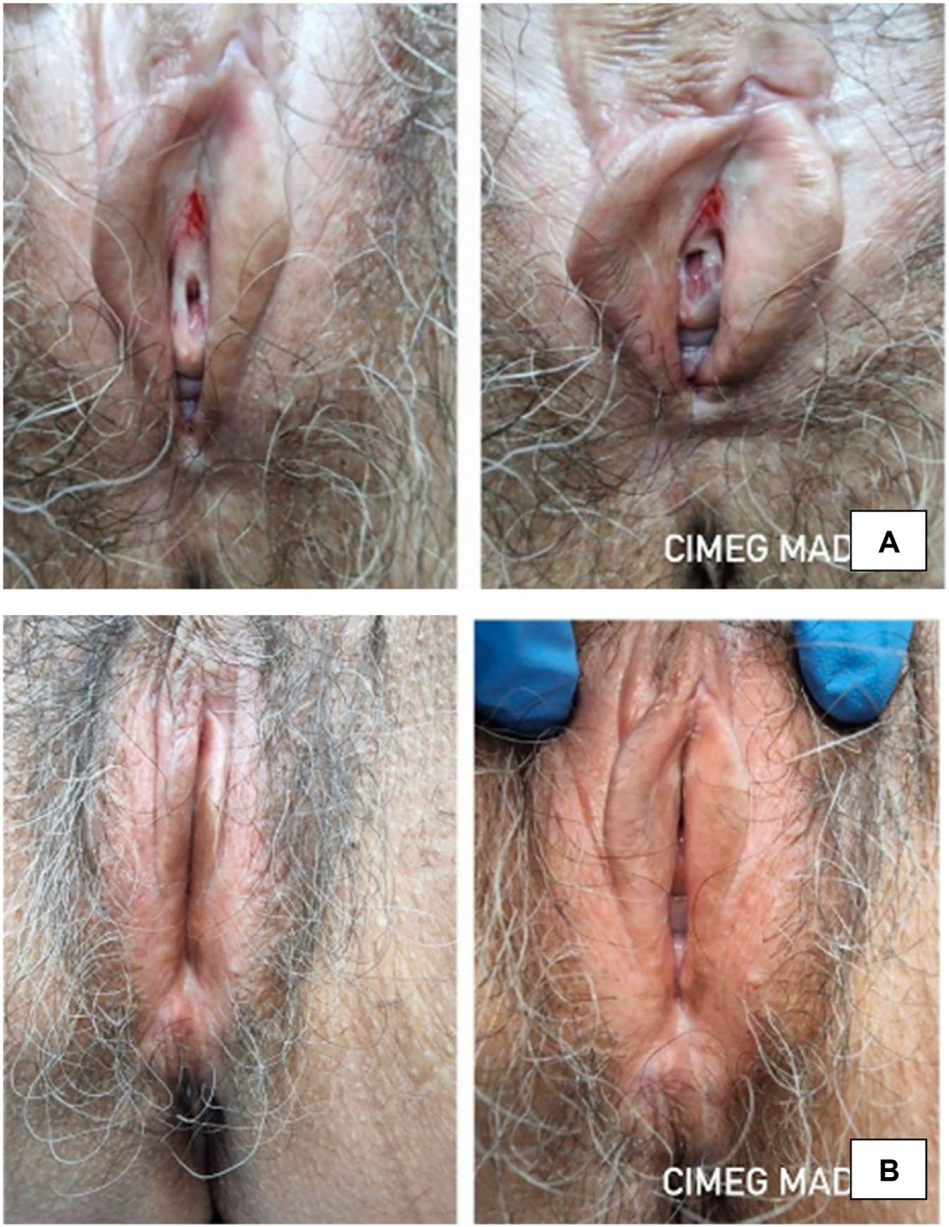
(A) Baseline images before treatment. (B) Posttreatment after two sessions of local fractional bipolar radiofrequency energy therapy with gold microneedles in combination with a topical exosome solution. Images provided courtesy of Dr. Zuramis Estrada Blanco.

A 60‐year‐old female patient presented with severe vulvar LS for 5 years despite prior treatment with topical corticosteroids and various moisturizers. Seeking other solutions, the patient was seen by a urogynecologist who recommended local fractional bipolar radiofrequency energy therapy with gold microneedles in combination with topical exosome therapy. Two sessions were performed. Prior to treatment, local anesthetic cream was applied to the entire vulva for 40 min and the area was cleaned. Immediately after each session, a topical exosome solution was applied to the vulvar area, including the vestibule, clitoral hood, labia minora, and majora. After the first session, she was able to incorporate the use of vaginal dilators, allowing her to resume sexual activity. The second session was performed 4 weeks after the first. After both sessions, there was a reduction in the appearance of depigmented atrophic patches and the patient noted improvement in pruritus and stinging sensations (Figure 16).

## 11 Exosomes in Systemic Sclerosis

Systemic sclerosis (SSc) is a chronic autoimmune disorder defined by progressive fibrosis of both the skin and internal organs. In SSc, exosomes play a role by releasing profibrotic messengers such as transforming growth factor‐beta (TGF‐β), interleukin‐6 (IL‐6), and metalloproteinases (MMPs) [43]. Altogether, these processes promote sclerosis. In SSc patients, exosomes also contain microRNAs (miRNAs) which regulate gene expression in favor of fibrosis [44]. Given that exosomes play a role in SSc pathogenesis, they could serve as a promising therapeutic target and potential biomarker for diagnosis, disease evolution monitoring, and treatment response. Exosome‐mediated transfer is another recently discovered mechanism which can induce therapeutic effects via mesenchymal stem cell transplantation. An investigation of mice with SSc demonstrated that the transfer of specific miRNA (miR‐151‐5p) from donor mesenchymal stem cell exosomes successfully induced immune tolerance by downregulating the IL4Rα/mTOR pathway in recipient bone marrow mesenchymal stem cells [45]. In the case of scleroderma, a murine study utilized exosomes derived from human umbilical cord mesenchymal stem cells to reduce extracellular matrix deposition, suppress epithelial–mesenchymal transition, and restore balance between M1 and M2 macrophages [46]. Given the proposed functions that exosomes serve in management of SSc among murine studies, next steps include further research regarding the use of exosomes in humans with SSc.

## 12 Exosomes in Systemic Lupus Erythematosus

Systemic lupus erythematosus (SLE) is an autoimmune condition that affects multiple organs, especially the skin and joints. In a study comparing exosomes purified from SLE patients versus healthy controls, it was found that exosomes in SLE patients contained higher levels of IFN‐α, TNF‐α, IL‐1β, and IL‐6, leading to the promotion of inflammation [47]. It was also identified that circulating exosome levels correlated with disease activity in SLE patients, making them a potential biomarker for disease activity. In addition to screening and disease monitoring, exosomes can be used to deliver anti‐inflammatory molecules, reducing the production of antibodies in SLE patients [44, 48, 49]. A study utilizing human umbilical cord mesenchymal stem cell‐derived exosomes injected intravenously into mice found that this intervention resulted in reduced proliferation of macrophages [50]. There was also increased splenic infiltration of regulatory T cells in these mice. Exosome administration alleviated nephritis and liver and lung injuries in the study population. Given this improvement in organ damage, exosome therapy may be a potential therapy that aids in improving the survival of patients with SLE. Investigations to improve large‐scale delivery and application of exosomes are underway [51].

## 13 Exosomes in Vitiligo

Vitiligo is an autoimmune disorder which causes loss of skin pigmentation [52]. Exosomes may play a role in repairing damaged keratinocytes while modulating the immune response [53]. Exosomes may also mitigate pigment changes in vitiligo by regulating gene activity involved in melanin production [54]. A study using a mouse model demonstrated the utility of human umbilical mesenchymal stem cell–derived exosomes in vitiligo [55]. This therapy demonstrated reduced skin depigmentation, less CD8+ T cell infiltration, and expanded regulatory T‐cell populations in skin. Similarly, there was reduced oxidative stress–induced melanocyte apoptosis. These effects were achieved via the delivery of specific microRNA (miR‐132‐3p and miR‐125b‐5p). Another study found that a specific microRNA (miR‐2909) pathway could be targeted to alter pathogenesis in vitiligo [56]. Exosomes isolated from healthy keratinocytes and fibroblasts were transfected into defective melanocytes. This revealed inhibited expression of miR‐2909 with restored melanocyte functioning and survival. Several preclinical studies have also demonstrated the promising results associated with mesenchymal stem cell–derived exosome therapies in vitiligo.

## 14 Limitations

Exosome therapy offers a compelling and unique treatment modality for a myriad of diseases, but several challenges and limitations hinder its clinical application. Notably, exosome therapy is not yet approved by the Food and Drug Administration (FDA) and remains largely unregulated [57]. This is likely tied to the absence of standardized protocols for the isolation, purification, and characterization of exosomes. Variability in production methods between research groups and manufacturing facilities results in inconsistencies in exosome quality, potency, and reproducibility.

While the production of MSC‐derived freeze‐dried exosomes has been validated as a good manufacturing practice (GMP)‐compliant process, concerns persist regarding their clinical translation [58]. Key unknowns, such as biodistribution, pharmacodynamics, and cellular fate following uptake, limit the development of precise dosing strategies and raise questions about long‐term safety [59, 60]. Analysis of these parameters is particularly challenging due to the nano‐sized nature and heterogeneous composition of exosomes, which complicates tracking and functional assessment.

Potential side effects and off‐target effects remain areas of concern. Although topical exosome applications are less prone to systemic risks due to their confinement to the nonliving stratum corneum, systemic delivery (e.g., intravenous, intramuscular, or intranasal) introduces uncertainties related to organ distribution and immune responses. Although preclinical safety studies have shown promising results, including a 2020 toxicology study that found no adverse effects from human adipose‐derived MSC exosomes in topical applications and animal studies without evidence of organ damage or inflammatory responses, comprehensive toxicological evaluations are still needed before exosomes can be confidently used in human medicine [61, 62]. Regulatory issues further complicate the pathway to approval, as the classification of exosomes (as a biologic, drug, or medical device) remains ambiguous, creating hurdles in navigating clinical trials and commercialization.

## 15 Conclusion

Despite these limitations, exosome therapy may hold significant potential in improving our treatment armamentarium for several diseases by offering targeted, cell‐free treatments with broad applications. Future research should prioritize addressing the challenges associated with standardization, safety, and regulatory approval. Developing robust, reproducible protocols for the isolation, characterization, and quality control of exosomes is critical to ensure their consistency and efficacy. Innovations such as advanced imaging techniques and molecular tagging could help elucidate biodistribution and cellular fate, providing crucial insights into their pharmacokinetics and pharmacodynamics.

Further toxicological studies and large‐scale clinical trials are essential to establish safety profiles across diverse populations and conditions. These investigations should focus on understanding potential immune responses, long‐term effects, and optimal dosing regimens for different routes of administration. In parallel, regulatory frameworks need to be streamlined to classify exosomes appropriately, ensuring clarity for researchers, manufacturers, and clinicians.

Emerging technologies, such as bioengineered exosomes and synthetic vesicle mimetics, offer novel directions for enhancing the therapeutic potential of exosomes. These advancements could enable more precise cargo loading, targeted delivery, and controlled release of exosomal contents. Additionally, multidisciplinary collaboration among scientists, clinicians, and industry stakeholders will be instrumental in translating exosome‐based therapies from the bench to the bedside.

With ongoing innovation and rigorous investigation, exosome therapy has the potential to transform dermatology and other fields by providing safe, effective, and personalized treatment options.

## Author Contributions

V.D., M.S., L.B., C.S., L.S., N.S., and T.S. all contributed to the planning, review, synthesis, and writing of the manuscript. All authors have read and approved the final manuscript.

## Conflicts of Interest

Victoria Dukharan, Milaan Shah, Luke Broughton, Carol Stegura, Luna Samman, and Nina Schur have no conflicts of interest to declare. Todd Schlesinger serves as a consultant, investigator, speaker, and/or advisor for Abbvie, Almirall, Allergan (An Abbvie company), ASLAN Pharma, Arcutis, Biofrontera, Beirsdorf, Benev, Bristol‐Myers Squibb, Castle Biosciences, Galderma, Eli Lilly, ExoCoBio, Incyte, Janssen, LEO, L'Oreal, Novartis, Pfizer, Regeneron, Sanofi, Sun Pharma, Takeda, UCB Pharma, and Verrica.

## Supporting information


Table S1.

## Data Availability

Data sharing not applicable to this article as no datasets were generated or analysed during the current study.
