# Supplementary material for: The Role of Exosomes in Medical Dermatology: Literature Review and Update
Source: J Cosmet Dermatol. 2025 Jan 10;24(1):e16761. doi: 10.1111/jocd.16761 (PMC11724262; doi:10.1111/jocd.16761)
Supplement: Supplementary file 1 — Table S1. [file JOCD-24-e16761-s001.docx]

**Supplement**

**Table 1: Summary of Exosome Applications and Outcomes in Dermatology**

| **Condition** | **Exosome Source** | **Application Method** | **Outcome** | **Key Study/Case Description** |
| --- | --- | --- | --- | --- |
| **Wound Healing** | ADSCs, iPSC-derived | Topical | Accelerates keratinocyte/endothelial migration, reduces inflammation, enhances re-epithelialization. | **Key Study:** Complete re-epithelialization in animal models.^18-22^ |
|  | Plant-derived | Topical | Complete closure of chronic non-healing axillary wound in 7 weeks. | **Clinical Case:** 24-year-old female with hidradenitis suppurativa. |
|  | Plant-derived | Topical | 90% closure of a third-degree ulcer with exposed muscle/bone in 26 days. | **Clinical Case:** 79-year-old female with traumatic scalp injury. |
|  | Plant-derived + Hyaluronic Acid Gel | Topical | Significant oral re-epithelialization in 24 hours, enabling oral feeding; lip healing by day 6. | **Clinical Case:** 16-year-old female with Stevens-Johnson syndrome. |
| **Flap Reconstruction** | ADSCs | Intradermal Injection | Improves skin flap survival rate, reduces scarring, promotes angiogenesis, and stimulates collagen and elastin production. | **Key Study:** Animal studies demonstrate efficacy.^23-26^ |
| **Radiation Dermatitis** | ESC-derived, MSC-derived, plasma-derived | Topical, Intravenous | Exosomal miRNAs (e.g., miR-126, miR-135a, miR-146) promote epithelial repair, protect against oxidative stress, and regulate immune responses. | **Key Study:** Studies highlight roles of miRNAs in epithelial migration, inflammation reduction, and antioxidant defense.^27^ |
|  | Plant-derived | Topical | Lightening of radiation-induced pigmentation in 3 weeks; additional lightening of surgical scars. | **Clinical Case:** 48-year-old female post-radiation therapy for breast cancer. |
| **Acne Vulgaris** | ADSCs | Intralesional | Inhibits inflammation (via reduced IL-1β and NLRP3 inflammasome) and NETs, reducing acne severity. | **Key Study:** Reduced inflammation and redness in mouse models.^29,30^ |
|  | Plant-derived | Intralesional with air dissector, RF microneedling, and a combined 589nm & 1319nm laser | Significant lesion improvement after 2 sessions over 2 months. Therapy was administered for four sessions every two weeks. | **Clinical Case:** 43-year-old male with severe acne vulgaris. |
|  | Plant-derived | Topical with picosecond laser | Accelerated post-laser scar healing; reduced redness and swelling within a week compared with laser treatment without exosomes. | **Clinical Case:** 25-year-old female with acne scars. |
|  | Plant-derived | Topical with picosecond laser | Enhanced healing of acne scars 3 months after a single session of picosecond laser compared to ceramide moisturizer. | **Clinical Case:** Comparison in a single patient post-laser treatment. |
| **Psoriasis** | MSC-derived, siRNA-loaded | Topical | Reduces IL-17/IL-23 levels, suppresses immune dysregulation, decreases scaling and erythema. | **Key Study:** Reduced lesions and scaling in murine models.^31,32^ |
|  | Plant-derived | Topical | Reduced scaling by 35 days and resolved erythema by 65 days. | **Clinical Case:** 30-year-old female with erythematous plaques. |
| **Atopic Dermatitis (AD)** | ADSCs | Topical, SubQ, IV | Reduces inflammatory cytokines, eosinophils, IgE levels; improves skin hydration and barrier function. | **Key Study:** Reduced inflammation and improved hydration in mouse models.^36-38^ |
|  | Plant-derived | Topical | Complete clearance of refractory lesions in 21 days, with no recurrence after 2 months. | **Clinical Case:** 6-year-old female with refractory AD. |
|  | Plant-derived | Topical | Reduced facial redness caused by dupilumab therapy; increased patient satisfaction. | **Key Study:** 12-week prospective study on dupilumab-associated facial redness.^39^ |
| **Allergic Contact Dermatitis (ACD)** | MSC-derived | Topical | Modulates T cell responses, reduces pro-inflammatory cytokines, increases Tregs and IL-10. | **Key Study:** Immune modulation demonstrated in mouse models of ACD.^41^ |
|  | Plant-derived | Topical | Resolution of pruritus, scaling, and redness in 14 days. | **Clinical Case:** 65-year-old male with exfoliative dermatitis due to ACD. |
| **Lichen Simplex Chronicus (LSC)** |  |  | Limited preclinical data on exosome therapy in LSC. |  |
|  | Plant-derived | Topical with microneedling | Reduced erythema, lichenification, and pruritus within 7 days; improved quality of life. | **Clinical Case:** 39-year-old male with LSC. |
| **Vulvar Lichen Sclerosus (LS)** |  |  | Limited preclinical data on exosome therapy in vulvar LS. |  |
|  | Plant-derived | Topical with RF Microneedling | Resumed sexual activity, reduced pruritus and atrophy after two sessions. | **Clinical Case:** 60-year-old female with severe vulvar LS. |
| **Systemic Sclerosis (SSc)** | MSC- derived, Umbilical cord-derived | Intravenous | Reduces fibrosis, promotes immune balance, and improves macrophage polarization. | **Key Study:** miRNA-loaded exosomes reduce fibrosis via IL4Rα/mTOR pathway in murine studies.^46, 47^ |
|  |  |  | No clinical cases reported yet. |  |
| **Systemic Lupus Erythematosus (SLE)** | Umbilical cord-derived | Intravenous | Reduces inflammation (ie, macrophage proliferation), expands regulatory T cells, improves organ function. | **Key Study:** Exosomes alleviate nephritis, liver damage, and cytokines in a murine study.^51^ |
|  |  |  | No clinical cases reported yet. |  |
| **Vitiligo** | Umbilical cord-derived, Keratinocyte-derived | Intravenous, direct melanocyte transfection | Reduces CD8+ T cell infiltration and oxidative stress, promotes melanocyte survival and function. | **Key Study:** Reduced depigmentation and enhanced melanocyte survival in mice.**^56,57^** |

| Abbreviations: Adipose derived stem cells (ADSC), induced pluripotent stem cells (iPSC), mesenchymal stem cell (MSC), small interfering RNA (siRNA), subcutaneous (SubQ), intravenous (IV), radiofrequency (RF). |
| --- |
